# Prediction of Cell Survival Rate Based on Physical Characteristics of Heavy Ion Radiation

**DOI:** 10.3390/toxics12080545

**Published:** 2024-07-27

**Authors:** Attila Debreceni, Zsolt Buri, István Csige, Sándor Bodzás

**Affiliations:** 1 Department of Mechanical Engineering, Faculty of Engineering, University of Debrecen, 4028 Debrecen, Hungary; 2 Department of Engineering Management and Enterprise, Faculty of Engineering, University of Debrecen, 4028 Debrecen, Hungary; 3 Karoly Ihrig Doctoral School of Management and Business, University of Debrecen, 4032 Debrecen, Hungary; 4 Department of Environmental Physics, Faculty of Science and Technology, University of Debrecen—ATOMKI, 4026 Debrecen, Hungary

**Keywords:** statistical learning, machine learning, ionizing radiation, heavy ion radiation, random forest, linear-quadratic model, local regression

## Abstract

The effect of ionizing radiation on cells is a complex process dependent on several parameters. Cancer treatment commonly involves the use of radiotherapy. In addition to the effective killing of cancer cells, another key aspect of radiotherapy is the protection of healthy cells. An interesting position is occupied by heavy ion radiation in the field of radiotherapy due to its high relative biological effectiveness, making it an effective method of treatment. The high biological efficiency of heavy ion radiation can also pose a danger to healthy cells. The extent of cell death induced by heavy ion radiation in cells was investigated using statistical learning methods in this study. The objective was to predict the healthy cell survival rate based on the physical parameters of the available ionizing radiation. This paper is based on secondary research utilizing the PIDE database. Throughout this study, a local regression and a random forest model were generated. Their predictions were compared to the results of a linear-quadratic model commonly utilized in the field of ionizing radiation using various metrics. The relationship between dose and cell survival rate was examined using the linear-quadratic (LQM) model and local regression (LocReg). An ***R*^2^** value of 88.43% was achieved for LQM and 89.86% for LocReg. Upon incorporating linear energy transfer, the random forest model attained an ***R*^2^** value of 96.85%. In terms of ***RMSE***, the linear-quadratic model yielded 9.5910^−2^, the local regression 9.2110^−2^, and the random forest 1.96 × 10^−2^ (lower values indicate better performance). All of these methods were also applied to a log-transformed dataset to decrease the right skewedness of the distribution of the datapoints. This significantly reduced the estimates made with LQM and LocReg (28% decrease in the case of ***R*^2^**), while the random forest retained nearly the same level of estimation as the untransformed data. In conclusion, it can be inferred that dose alone provides a somewhat satisfactory explanatory power for cell survival rate, but the inclusion of linear energy transfer can significantly enhance prediction accuracy in terms of variance and explanatory power.

## 1. Introduction

Understanding the physiological role of radiation holds extreme importance given its potential benefits and hazards. Among these, ionizing radiation assumes a particularly significant role physiologically, primarily due to its profound cell-destructive effects [1]. Although the broad implications of ionizing radiation on cellular destruction are well-known, accurately predicting the extent of cell death remains immensely challenging, even with various biological and radiation parameters such as cell type, state, cycle, radiation type, energy, delivered dose, and irradiation duration. The complexity arises from the multitude of parameters whose roles are not always clearly defined and vary case by case. Additionally, radiation can induce deterministic or stochastic effects, further contributing to uncertainties in the overall scenario [2]. The objective of this study was to construct statistical models, commonly employed in various disciplines, and assess them based on available heavy ion ionizing radiation data. These models were subsequently compared with the frequently utilized linear-quadratic model in the literature [3]. The ionizing radiation data utilized were sourced from the Particle Irradiation Data Ensemble (PIDE) database compiled by the GSI Helmholtzzentrum für Schwerionenforschung. Based on this foundation, conclusions could be drawn regarding the reliability, complexity, as well as the advantages and disadvantages of the models developed.

## 2. Materials and Methods

### 2.1. Radiation Parameters

To investigate the physiological impacts of ionizing radiation, it becomes imperative that various specific properties be quantified. Radiation assessment relies on multiple characteristics. One fundamental measure is the absorbed dose, which can be directly or indirectly applied to ionizing radiation. The absorbed dose indicates the energy “***E***” transferred by the radiation to a unit mass “***m***” within a specified volume [4,5]:

(1) $$D=\frac{d\underline{E}}{dm}\;[\frac{J}{\mathrm{kg}}=\mathrm{Gy}]$$

where

***dE*** is the radiant energy absorbed by the material;

***dm*** is the mass of the material.

The effect of ionizing radiation on a given tissue can be influenced by another characteristic known as linear energy transfer (***LET***), which quantifies the amount of energy “***E***” transferred by the radiation per unit distance “***l***” within the tissue [4,5]:

(2) $$LET=\frac{dE}{dl}\;[\frac{\mathrm{keV}}{\mu m}]$$

In the case of a high linear energy transfer, a large amount of energy is lost by the ionizing radiation over a given distance, so it typically cannot penetrate as deep into the tissue as a lower ***LET***. From the point of view of local tissue damage, radiation with a high linear energy transfer has a high tissue-damaging effect [4,5].

Carbon heavy ion radiation was first utilized as a radiotherapy treatment in Japan in 1994 [6]. The advantage of heavy ion radiotherapy treatments is that high energy can be delivered to the target tissue with high precision, while only a small amount of energy is delivered away from the target tissue [7]. The energy delivered by heavy ion beams can be characterized by a Bragg curve showing the amount of linear energy transfer (LET) and dose that occurs as a function of the distance traveled in the tissue. Consequently, significant cell killing occurs where the Bragg peak is located, making it effectively possible to treat cancer cells [8,9].

### 2.2. Supervised Learning

In supervised learning, predictions are made by constructing a dataset from existing data that have been collected and selected [10]. A distinguishing feature compared to unsupervised machine learning is that while both methods involve datasets with input independent variables, output dependent variables are only required for supervised machine learning. The name is derived from this fact, as the output variables are already known to “supervise” the parameterization of the model [11]. The resulting dataset is divided into a training dataset and a test dataset according to some chosen ideal method. Such a method could be hold-out validation, cross-validation, leave-p-out cross-validation, etc. [12]. Using the training dataset, the statistical model is trained and is capable of making predictions for data points that were not included in the training dataset. The purpose of the test dataset is to assess the accuracy of the predictions made by the generated and trained model [13]. A common example of supervised learning is the prediction of the selling price of real estate based on the dimensions of its floor plan or the grouping of animals of different species according to their appearance (body length, body height, wingspan, etc.). For supervised learning problems, the two main approaches are classification and regression. In the course of this study, algorithms and evaluation methods commonly used in the field of statistical learning theory were used [14]. 

### 2.3. Compilation of Datasets

The data for this study were sourced from the PIDE database, which was established by the GSI Helmholtz Centre for Heavy Ion Research (GSI Helmholtz-Zentrum für Schwerionenforschung). The database comprises in vitro measurements derived from biological reactions induced by ionizing irradiation across 1118 experiments. These measurements were compiled from prior publications, with the oldest dating back to 1975 and the most recent to 2015 [15]. The database is comprehensive, encompassing results from irradiation with various heavy media. Numerous characteristics are enumerated, such as cell line, heavy ion, linear energy transfer, dose, etc. This study explores the effect of physical parameters of irradiation; thus, the database has been filtered based on biological characteristics. The NB1RGB cell line was selected for the database filtering. This cell line was chosen because it is of human origin, increasing the potential relevance of the research results. Additionally, the PIDE database contains numerous data points for this cell line, with 51 out of 202 experiments on healthy human cell lines with asynchronous cell cycles using the NB1RGB cell line. Therefore, the primary reason for selecting this cell line is statistical. At the measurement level, this action reduces the database to 51 experiments. Within these 51 measurements, a total of 318 data points were contained. A medium-sized dataset, consisting of 318 data points, was created. For a dataset of this size, several validation methods are available. Hold-out validation can be used to generate the training and test datasets, which, for such a large dataset, can produce results similar to the cross-validation method but requires less computational power. On the other hand, more complex methods such as cross-validation can produce better results at the expense of greater computational power [16,17]. These 318 data points constitute the entire dataset from which the training and test datasets were made via the Monte Carlo cross-validation procedure. This procedure splits the dataset into training and test subsets with a random ratio over many iterations. One hundred was chosen as the number of iterations. This allows real empirical measurement data to be used for validating the models. After filtering, the following features were retained as independent variables: dose, ion type, charge, linear energy transfer, and energy. Since the charge of the ion is dependent on the nucleus, the ion type and charge features are not independent of each other, making it impractical to consider both in the learning process [18]. 

**Figure 1:**
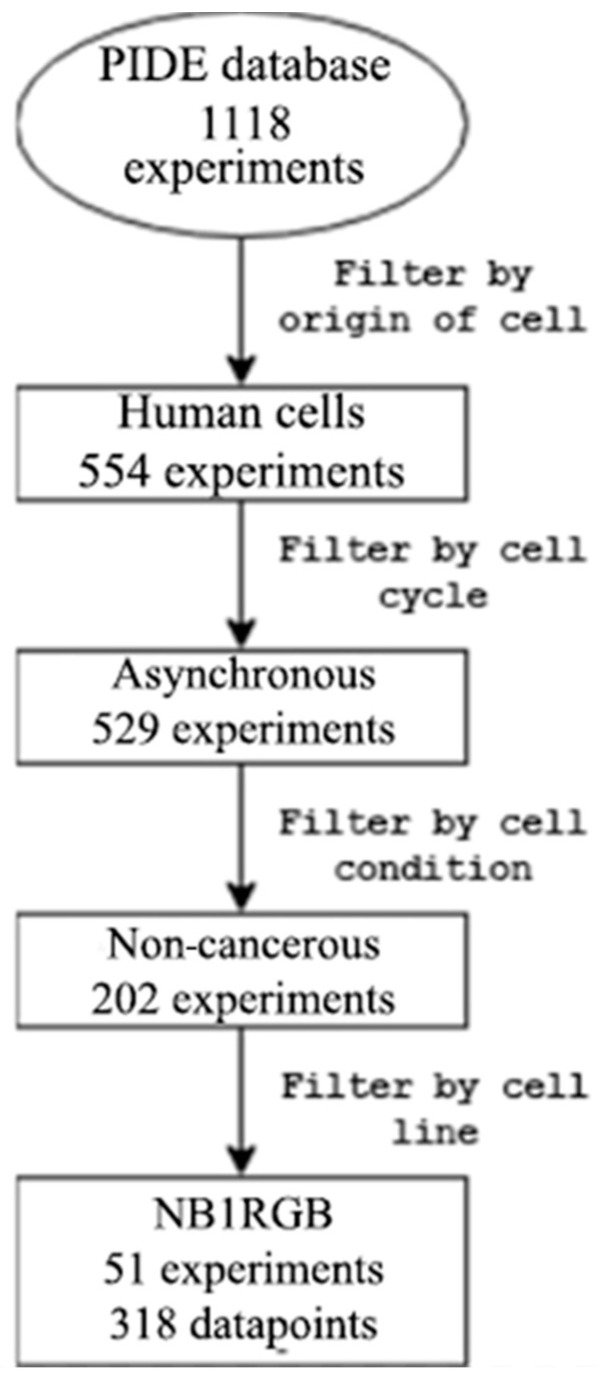
Experiments in the PIDE database were filtered by biological characteristics.

In the filtered database, there were four isotope types: 12C, 20Ne, 28Si, and 56Fe. Among the 51 experiments, 24 utilized 12C isotopes, 15 employed 20Ne, 7 utilized 28Si, and 5 utilized 56Fe. The prominent role of the 12C isotope among the heavy ions is demonstrated by these data. One of the most popular uses of heavy ion radiotherapy is attributed to the use of carbon ions [19,20]. Figure 1 shows the steps of filtering based on the biological features.

### 2.4. Applied Methods

The linear-quadratic model is the mathematical equation for ionizing radiation, which establishes a mathematical relationship between dose and cell survival rate [21,22]:

(3) $$S=e^{-\alpha \times D-\beta \times D^{2}}$$

where

***S*** is the cell survival rate [%];

***D*** is the dose [Gy];

***α*** is the linear weight parameter;

***β*** is the quadratic weight parameter.

In general, it is observed that at low doses, a larger role is played by the linear α factor, while the role of the quadratic ***β*** factor increases proportionally as one moves towards higher doses and becomes decisive at higher doses. The roles of the linear and quadratic terms in the equation can be attributed to biological phenomena, specifically the interaction between radiation and cellular DNA [21]. According to an early hypothesis, the parameter α can be derived for the damage caused by an ionizing particle to both strands of DNA in a double helix at the same time. Conversely, the ***β*** parameter causes damage at multiple sites, ultimately resulting in double-strand breaks [23]. Later, other explanations emerged, such as the suggestion by Goodhead that the rate of cell repair decreases as the number of lesions increases [24].

The random forest model is an ensemble machine learning model that combines several decision tree models to produce a stronger and more stable prediction [25]. It is a commonly used and powerful technique in machine learning, especially for achieving higher accuracy and generalizability. When a random forest model is created, the decision trees are based on different baseline learning datasets. These tree models differ from each other because they learn on randomly selected subsets of datasets. With this method, the models are diversified, reducing overfitting and variance error [26]. The way the model works is that each tree generates an individual prediction, and then these predictions are combined by majority voting or averaging, for example, to make the final decision. This process reduces the risk of overfitting while improving the overall performance of the model [27]. On the one hand, bootstrapped datasets are used by decision trees; an extremely important feature of these datasets is that the same data point can be extracted multiple times from the base training dataset. This is to ensure that the resulting random forest model is not sensitive to any single data point. This method is called bagging [28]. 

Local regression is an extension of linear regression, which allows non-linear relationships to be understood with high precision by the model, thus providing predictions of sufficient quality [29]. The essence of local regression is that a straight line is not fitted to the entire training dataset, but locally weighted models with a smoothing function are created and summarized. The weighting function applied to these models is a so-called window function, also known as a kernel. The role of the kernel is such that, when forming the prediction, only data within the range specified by the kernel are considered by the local model to be trained, and the data within this range are weighted by the weight function [28,30].

## 3. Results

### 3.1. Cell Survival Rate and Dose Relationship

**Figure 2:**
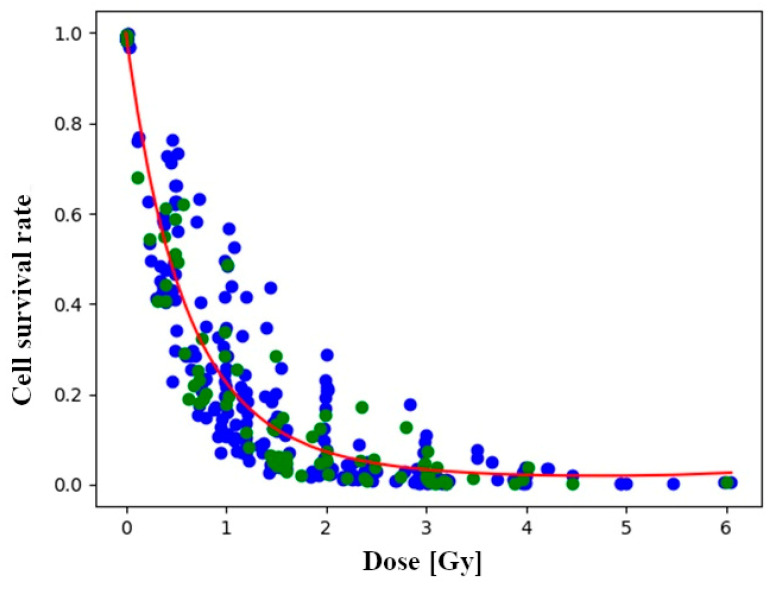
Fitted linear-quadratic equation. The blue data points belong to the training dataset and the green ones to the test.

Figure 2 displays the fitting of the linear-quadratic model to the dataset. The values of the model coefficients ***α*** and ***β*** are dependent on the training dataset, so there may be a small to large difference in their values due to the given train-test split. As random Monte Carlo cross-validation train-test splitting can induce variation in the modeling process, the models are created 100 times, and their results are examined by averaging the 100 iterations of the model. The Monte Carlo cross-validation will use the datapoints unevenly; therefore, some degree of weighting of specific data points will occur [31]. Upon examining the results in Table 1, it can be concluded that the values of the ***α*** and ***β*** factors are to some extent dependent on the train-test split, with some degree of variance. 

**Figure 3:**
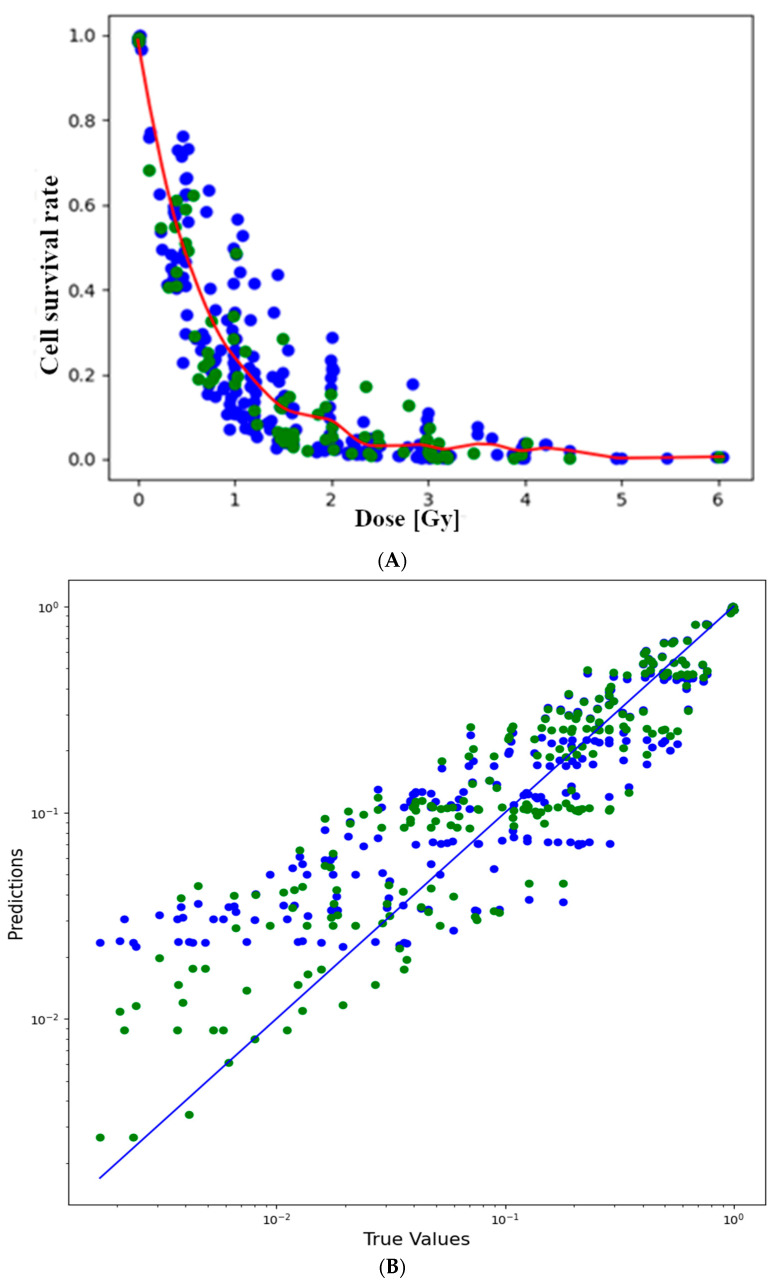
(**A**) Local regression curve. The blue data points belong to the training dataset and the green ones to the test. (**B**) Plot of predicted and actual values. The blue data points belong to the linear-quadratic model and the green ones to local regression.

The linear-quadratic model was compared with a local regression model. Figure 3B depicts the difference between the predicted values from the true values. If the dots are closer to the 45-degree line, the prediction is improved [32]. A broadly similar pattern was observed for both models. In the case of higher survival rates, the predictions were closer to the true values than in the case of lower survival rates. What is noticeable is that the linear-quadratic model is a poor predictor at very low survival rates (***S*** < 2%). Figure 2 also indicates that the curve of the linear-quadratic model has a rising edge, which strongly suggests poor prediction. This may be attributed to the fact that the linear-quadratic model took the full dataset into account during training. The models were evaluated using selected metrics such as the ***R*^2^** score and ***RMSE***. The ***R*^2^** score was the first to be examined, as it is the most consistent measure of the relationship between the output and input variables [33]. Upon examination of the coefficient of determination across 100 different train-test splits, it was observed that, on average, slightly better predictions were produced by local regression compared to the linear-quadratic model. The average coefficient of determination for local regression was recorded as ***R^2^_lr_*** = 0.8986, whereas for the linear-quadratic model, it was ***R^2^*_lqm_** = 0.8843. The difference in magnitude between the two averages is noted to be 0.0143. This discrepancy suggests that, on average, the cell survival rate is explained 1.43% more effectively in relation to dose by local regression [34]. Consequently, there appears to be no significant improvement in the explanatory power of dose. Notably, in the case of the linear-quadratic model, a higher degree of variance and dispersion, alongside a greater coefficient of variation in terms of metric, were observed as a result of the train-test split. Consequently, it was deemed to be more sensitive to the composition of the training and test datasets in terms of prediction compared to local regression.

**Figure 4:**
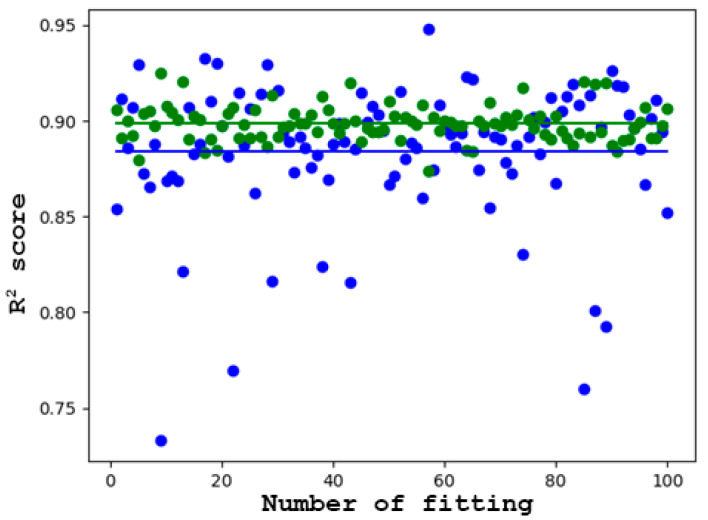
Evaluation of linear-quadratic model and local regression using the ***R*^2^** measure on a test dataset of 100 different compositions. The green dots are for the local regression and the blue dots are for the linear-quadratic model. The continuous lines illustrate the averaged ***R*^2^** values.

It is worth highlighting that while better coefficients of determination were yielded by the linear-quadratic model in certain instances, it performed significantly worse in others. This can be seen from Figure 4 and the variance data in Table 2. Additionally, the value of the standard deviation indicates that the correlation of the linear-quadratic model was more susceptible to variations in the dataset compared to local regression.

It is worth considering that since the ***MSE*** value is averaged over the square of the errors and, given that the errors are inherently small (as both the actual and predicted survival rates range between 1 and 0), squaring them yields exceedingly diminutive values, which may lead to potential misinterpretation. An advantage of ***RMSE*** over ***MSE*** is that it is similar in magnitude to the data. Therefore, the root mean square error (***RMSE***) value emerges as a more reliable metric for expressing the degree of error in the model [35,36].

The linear-quadratic model exhibited a mean ***RMSE*** that was 4.1% higher, along with a standard deviation that was 2.93 times greater and a coefficient of variation of 2.886, indicating that the reliability of the linear-quadratic model was more significantly impacted by the data points.

**Figure 5:**
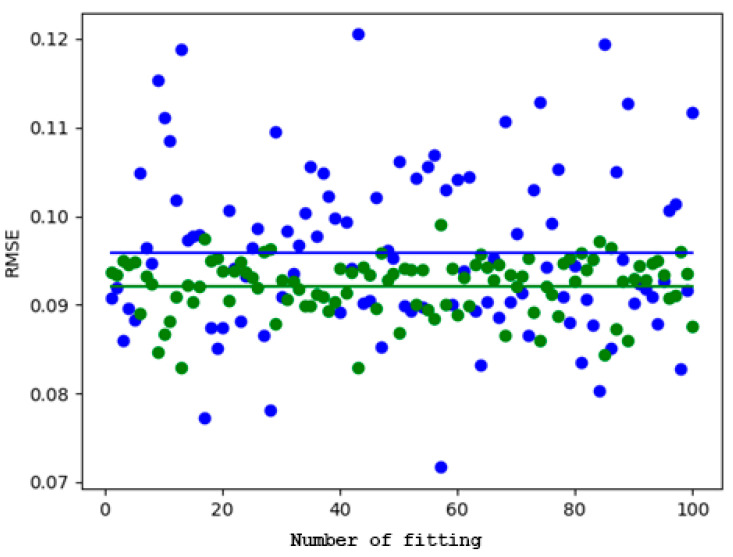
Evaluation of linear-quadratic model and local regression using ***RMSE*** measure on 100 test datasets of different compositions. Green dots are for the local regression, while blue dots are for the linear-quadratic model. The continuous lines represent the averaged ***RMSE*** values.

Summarizing these results in Table 3 and Figure 5, it can be concluded that better results are achieved by local regression compared to the linear-quadratic model. Although there is no significant increase in the explanatory power of the dose, with only a 1.43% difference between the ***R*^2^** values, a much smaller standard deviation in favor of local regression was observed. These findings also mostly apply to the ***RMSE***.

### 3.2. Fitting to the Natural Logarithm of the Cell Survival Rate

**Figure 6:**
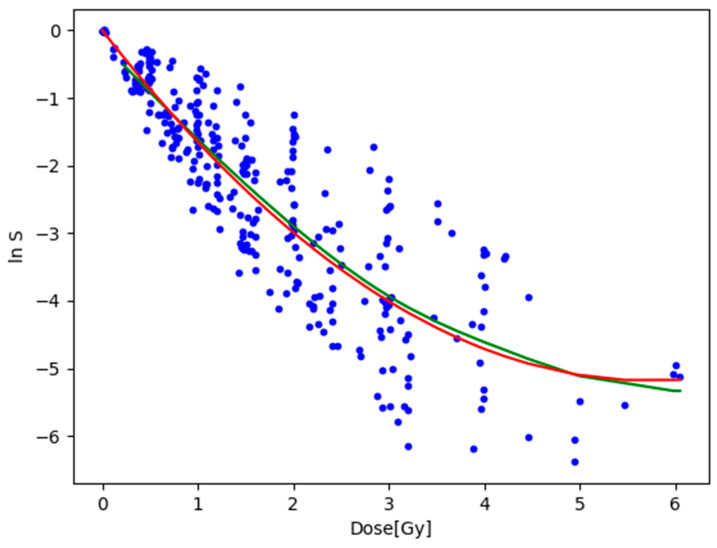
Results of fitting linear-quadratic and local regression models to the log-transformed data. Blue dots are the datapoints, red line represents linear-quadratic model, and green line represents local regression model.

Since the dataset has an exponential distribution, the ***R*^2^** value can be misleading. Consequently, the dataset was logarithmically transformed using a natural log transformation. The linear, quadratic, and local regressions were fitted again to the transformed dataset. The result of the fitting is shown graphically in Figure 6. Other parameters of the fit had not been changed. Upon examining the ***R*^2^** value in Table 4 of the fits, a significant 28% reduction was observed. 

**Figure 7:**
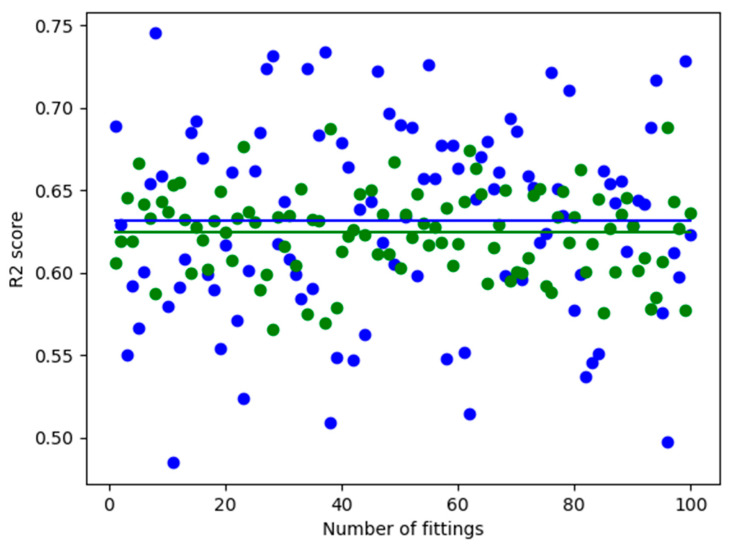
Evaluation of linear-quadratic model and local regression using ***R*^2^** measure on 100 log-transformed test datasets of different train-test splits. Green dots are for the local regression, while blue dots are for the linear-quadratic model. The solid lines represent the averaged ***RMSE*** values.

The comparison of the value of the ***RMSE*** with the range of ln S indicated that a ratio of around 12% existed for both models. This suggests significant variance, which can be readily observed by examining the fitted lines and the positioning of the data points. A somewhat interesting observation is that, when fitted to the transformed data, the linear-quadratic model slightly outperformed the local regression model. This difference amounted to only 1.1%, which is negligible. In summary, the results indicate that the local regression model does not offer a superior relationship between dose and cell survival rate compared to the traditional linear-quadratic model. Figure 7 demonstrates that the linear-quadratic model exhibited greater sensitivity to train-test split compared to local regression. For reference, a simple linear regression was fitted to the transformed dataset. The linear regressor fitted to the log-transformed data could be seen as equivalent to the linear no-threshold model due to its linear nature and consideration of small doses. Upon comparing its results with the other two models, a clear advantage was evident in favor of both the local regression and the linear-quadratic models, yielding an ***R*^2^** value of 14%. Therefore, it is apparent that utilizing the originally investigated models would result in significantly better predictions.

### 3.3. The Role of Linear Energy Transfer in the Rate of Cell Death

**Figure 8:**
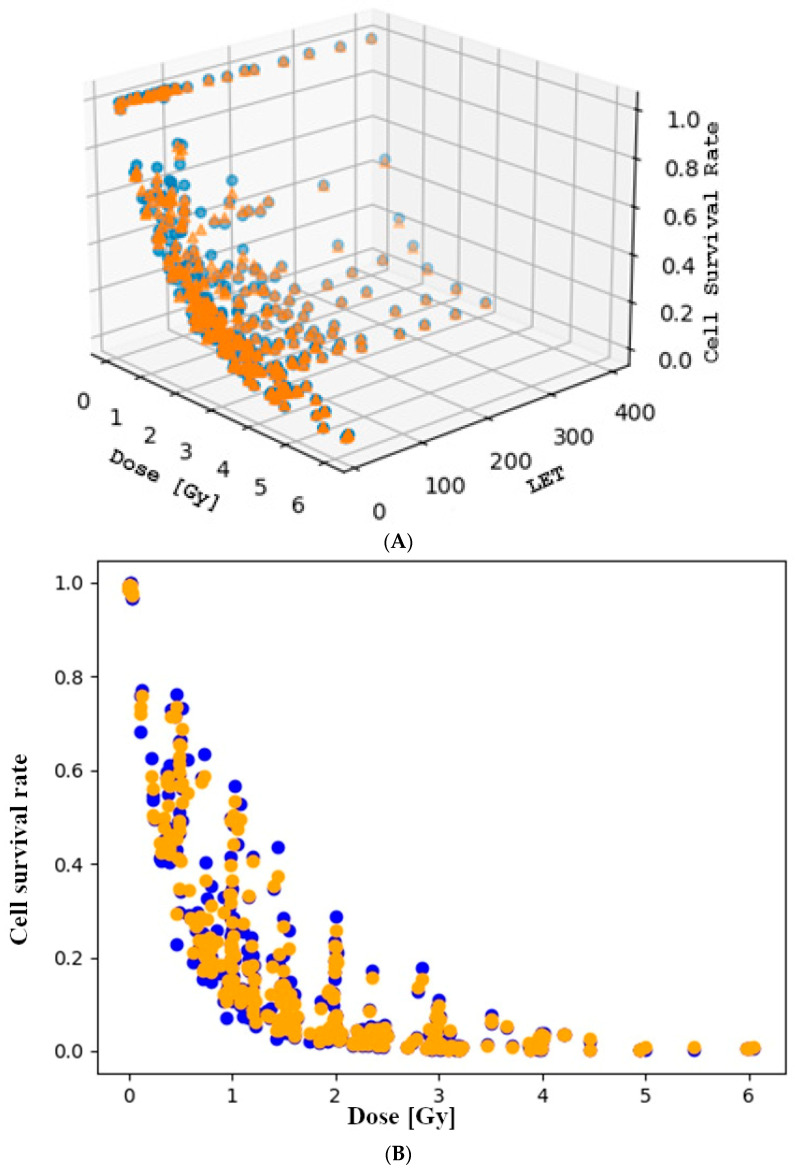
(**A**) Cell survival rate as a function of dose and linear energy transfer in 3D plot. (**B**) Cell survival rate as a function of dose and linear energy transfer in 2D plot. The blue data points are the complete dataset, while the orange ones are the predicted arteries of the model.

The third method utilized after the linear-quadratic model and local regression involved the application of a regression random forest model on the entire dataset. In addition to dose, another input feature, linear energy transfer, was incorporated into this model. The reason linear energy transfer was chosen is that it has been demonstrated in previous research to be an important parameter [37,38,39]. The parameterized random forest model initiated the generation of 1000 decision trees and subsequently applied regression prediction. To create the trees, a bootstrapped dataset was utilized, wherein randomly selected data points from the complete dataset were employed to train the decision trees [40]. Although each tree utilized the same number of data points as the total dataset, it is noteworthy that a data point could potentially be selected multiple times. Furthermore, the values of various metrics for evaluating the random forest performance were computed by the model using the out-of-bag data points, which were omitted during bootstrapping [41]. The train-test split was utilized to create a test dataset. The parameters of the random forest model were tuned using the grid search method in order to find the best performing parameter settings. The best performing settings by coefficient of determination can be examined in Table 5 and Table 6. Figure 8 shows the random forest model that was fitted with the best performing settings.

For the coefficient of determination, local regression was outperformed by the random forest by 0.07 and the linear-quadratic model by 0.084. This indicates that the introduction of linear energy transfer explained 7–8% more of the variation in cell survival rate. In the case of ***RMSE***, it may be more appropriate to consider the ratios rather than the differences. The ***RMSE*** of the random forest is 49 times smaller than that of the linear-quadratic model, while it is 47 times smaller than that of the local regression. Thus, the predicted values are much closer to the measured results with the introduction of linear energy transfer and the application of the random forest model. 

### 3.4. Comparison of the Performances in the Case of the Log-Transformed Dataset

To further investigate the random forest, the hyperparameters of the model were examined concerning the ***R*^2^** value of the estimate. The aim was to determine the extent to which changes in the hyperparameters affected the ***R*^2^** value used to evaluate the estimate. The ***R*^2^** of the estimate was not significantly affected by the hyperparameters of the random forest. The largest value was 0.9685, while the smallest was 0.9385, representing a relative difference of 1.34%. The OLS (ordinary least squares) method was used to further evaluate the models. The OLS model’s ***R*^2^** value of 0.828 indicated that approximately 82.8% of the variance in the dependent variable (***R*^2^** score) was explained by the model, suggesting a good fit. Similarly, the adjusted R-squared value of 0.820, adjusted for the number of predictors in the model, was slightly lower than the R-squared but still indicated a good fit, as it explained approximately 82% of the variance in the dependent variable. The ***F***-statistic (114.0) and its **p**-value (2.21 × 10^−35^) indicated that the model was statistically significant overall, suggesting that at least one of the predictors was significantly related to the ***R*^2^** score. Table 7 and Table 8 show the 5 best and worst performing parameter settings on the log transformed dataset.

The relationship between the random forest parameters and ***R*^2^** is shown in Table 9. In terms of statistical significance, n_estimators (1.2640 × 10^−7^, ***p*** = 0.836) and max_depth (5.17 × 10^−6^, ***p*** = 0.403) showed no significant impact on the ***R*^2^** score, with *p*-values exceeding 0.05. Conversely, min_samples_split (−0.0007, ***p***= 1.2 × 10^−22^) and min_samples_leaf (−0.0025, ***p***= 4.2 × 10^−30^) exhibited statistically highly significant coefficients, indicating a negative association with the ***R*^2^** score. Specifically, increasing the minimum number of samples required to split an internal node (min_samples_split) or to be at a leaf node (min_samples_leaf) significantly reduced the ***R*^2^** score. 

**Figure 9:**
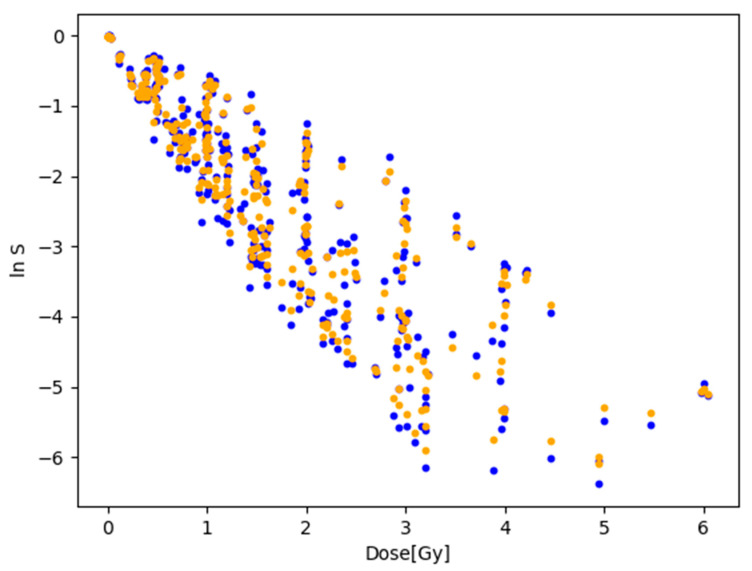
Prediction of random forest on the log-transformed dataset.

The comparison of the best performing random forest model with the previously established models is presented in Table 10. The random forest model is illustrated in Figure 9.

The tuned models were tested on other cell lines (T1, HFL-III, AG01522, HF19), with the bulk of the dataset constituted by the T1 cell line. The aim was to evaluate the applicability of the tuned models on other cell lines. The results are presented in Table 11.

Looking at the data recorded in the table, no significant reduction in the metrics for LQM was observed. The coefficient of determination decreased by 0.0058, indicating that the tuned model lost 0.58% of its explanatory value for a new cell line. Although an increase in ***RMSE*** was detectable, it was not significant. This suggests that the model can be applied to different cell line types without a significant deterioration in the metrics. For random forests that include LET as an input variable, the model’s explanatory power was already more sensitive to changes in cell line type, with an approximately 5.3% decrease observed in the coefficient of determination. An increase in ***RMSE*** by approximately two times was also noted, indicating a more significant level of unreliability for the model. These results may suggest that the same tuned model can be applied more flexibly to different types of cell lines without knowledge of the LET than one that already includes the linear energy transfer value. This concept can be further tested by introducing new models or extending existing ones.

## 4. Discussion

Through the comparison of results, it becomes feasible to quantify the impacts of ionizing radiation on cells, which can be adequately modeled, in principle, by the conventional linear-quadratic model. A remarkably strong correlation between the dose-based linear-quadratic model and the cell survival rate was indicated by an ***R*^2^** value of 0.88. It was suggested by the root mean square error (***RMSE***) value of 0.096 that the average prediction of the model deviated from the true value by approximately 9.6%. In contrast, somewhat superior outcomes compared to the linear-quadratic approach were yielded by the application of local regression to the dataset, particularly when examined in terms of doses. The cell survival rate was elucidated more effectively by 1.43%, as per the coefficient of determination, while the ***RMSE*** values exhibited a smaller mean error of 0.38% upon comparison. Overall, there appears to be no noteworthy distinction between the two modeling methodologies. It is noteworthy that the models were built using only 318 data points, which may not qualify as a large dataset. Consequently, it is conceivable that the marginal advantages favoring local regression might entirely vanish with a larger dataset. Notably, markedly improved results were yielded by the random forest model. This enhancement is primarily attributable to the inclusion of linear energy transfer as an independent input variable. With an almost 97% coefficient of determination value, it was demonstrated to be a highly suitable model for predicting the cell-destroying effect of radiation. For the other metrics, results can be provided that are one order of magnitude better than those obtained from the local regression and linear-quadratic models. The conclusions drawn in other studies are consistent with these results [37,39]. When considering the structure and complexity of the models, it is evident that the linear-quadratic model is the simplest. Additionally, it is the model that most directly expressed the relationship between dose and cell survival rate, and therefore, it is the easiest to interpret. The random forest model is the most complicated in principle and did not provide any level of mathematical correlation between dose and cell death induced by linear energy transfer. When fitting to a logarithmically transformed dataset, a more significant difference was observed between the random forest and the linear-quadratic models. For LQM and local regression, ***R*^2^** was reduced by almost 0.25. No similar reduction was observed for the random forest. The evaluation of models already tuned on the NB1RGB cell line on other cell lines concluded that a model based on dose alone could be confidently applied to other cell lines; a model using LET and dose alone reduced the accuracy of the estimation to a more significant extent, but it was still more accurate than a model using dose alone. Authors selected a specific experimental cell line. It is future work to verify the model using in vitro cells and expand to other cell types.

## Figures and Tables

**Table 1:** Result of fitting a linear-quadratic model 100 times.

	*α Coefficient*	*β Coefficient*
***Mean (AM)***	1.6939	−0.1827
***Variance***	0.0154	0.00178
***Standard deviation***	0.041	0.0137
***Minimum***	1.5804	−0.2177
***Maximum***	1.8257	−0.1281

**Table 2:** Comparison of linear-quadratic model and local regression ***R*^2^** results.

	*LQM*	*Local Regression*
***Mean(AM)***	0.8843	0.8986
***Variance***	0.03614	0.00924
***Minimum***	0.7329	0.8738
***Maximum***	0.9477	0.9248
***CV***	0.04087	0.01028

**Table 3:** Comparison of linear-quadratic model and local regression ***RMSE*** results.

	*LQM*	*Local Regression*
***Mean(AM)***	0.0959	0.0921
***Variance***	0.009382	0.0032
***Minimum***	0.0717	0.08286
***Maximum***	0.121	0.0986
***CV***	0.198	0.09901

**Table 4:** Comparison of the original linear-quadratic model and local regression results with the new results based on the transformed dataset.

	*LQM*	*Local* *Regression*	*LQM* *ln*	*Local* *Regression ln*	*Linear Regression ln*
***R^2^***	0.8843	0.8986	0.6316	0.6245	0.5531
***RMSE***	0.0959	0.0921	0.7689	0.76174	0.8952

**Table 5:** Result of hyperparameter tuning. The tuning was made with the grid search method.

*n_* *Estimators*	*Min_* *Samples_Split*	*Min_* *Samples_Leaf*	*Max_* *Depth*	*R^2^* *Score*	*RMSE* *Score*
1000	2	1	100	0.9685	0.0196
200	2	1	100	0.9511	0.129
600	2	1	100	0.9511	0.130
800	2	1	60	0.9509	0.131
400	2	1	100	0.9509	0.130

**Table 6:** Evaluation of linear-quadratic, local regression, and random forest models.

	*LQM*	*Local Regression*	*Random Forest*
***R^2^***	0.8843	0.8986	0.9685
***RMSE***	0.959	0.921	0.0196

**Table 7:** Result of hyperparameter tuning. The tuning was made with the grid search method. The table shows the 5 best performing hyperparameters.

*n_* *Estimators*	*Min_* *Samples_Split*	*Min_* *Samples_Leaf*	*Max_* *Depth*	*R^2^* *Score*	*RMSE* *Score*
200	2	1	40	0.941318	0.132077
600	2	1	20	0.940821	0.130237
400	2	1	40	0.940702	0.130339
800	2	1	100	0.940699	0.130532
1000	2	1	60	0.940498	0.131342

**Table 8:** Result of hyperparameter tuning. The tuning was made with the grid search method. The table shows the 5 worst performing hyperparameters.

*n_* *Estimators*	*Min_* *Samples_Split*	*Min_* *Samples_Leaf*	*Max_* *Depth*	*R^2^* *Score*	*RMSE* *Score*
800	10	4	80	0.92887905	800
600	10	4	20	0.928749352	600
600	10	4	100	0.928730675	600
400	10	4	40	0.928686579	400
200	2	4	80	0.928535596	200

**Table 9:** Hyperparameter coefficients and *p*-values.

	*Coefficients*	*p-Value*
n_estimators	1.264 × 10^−7^	0.836
min_samples_split	−0.0007	1.2 × 10^−22^
min_samples_leaf	−0.0025	4.2 × 10^−30^
max_depth	5.17 × 10^−6^	0.403

**Table 10:** Evaluation of linear-quadratic, local regression, and random forest models on the transformed dataset.

	LQM ln Transformed	Local Regression ln Transformed	Random Forest ln Transformed
***R^2^***	0.6316	0.6245	0.9413
***RMSE***	0.7689	0.76174	0.1321

**Table 11:** Results of tests of tuned models on different cell lines.

	LQM ln Transformed *R^2^*	Random Forest ln Transformed *R^2^*	LQM ln Transformed *RMSE*	Random Forest ln Transformed *RMSE*
NB1RGB	0.6316	0.9413	0.7689	0.1321
Other	0.6258	0.8884	0.9957	0.2708
Difference	−0.0058	−0.0529	0.2268	0.1387

## Data Availability

PIDE database used in the research is available at the following website: https://www.gsi.de/work/forschung/biophysik/forschungsfelder/radiobiological_modelling/pide_project accessed on 24 July 2024.
